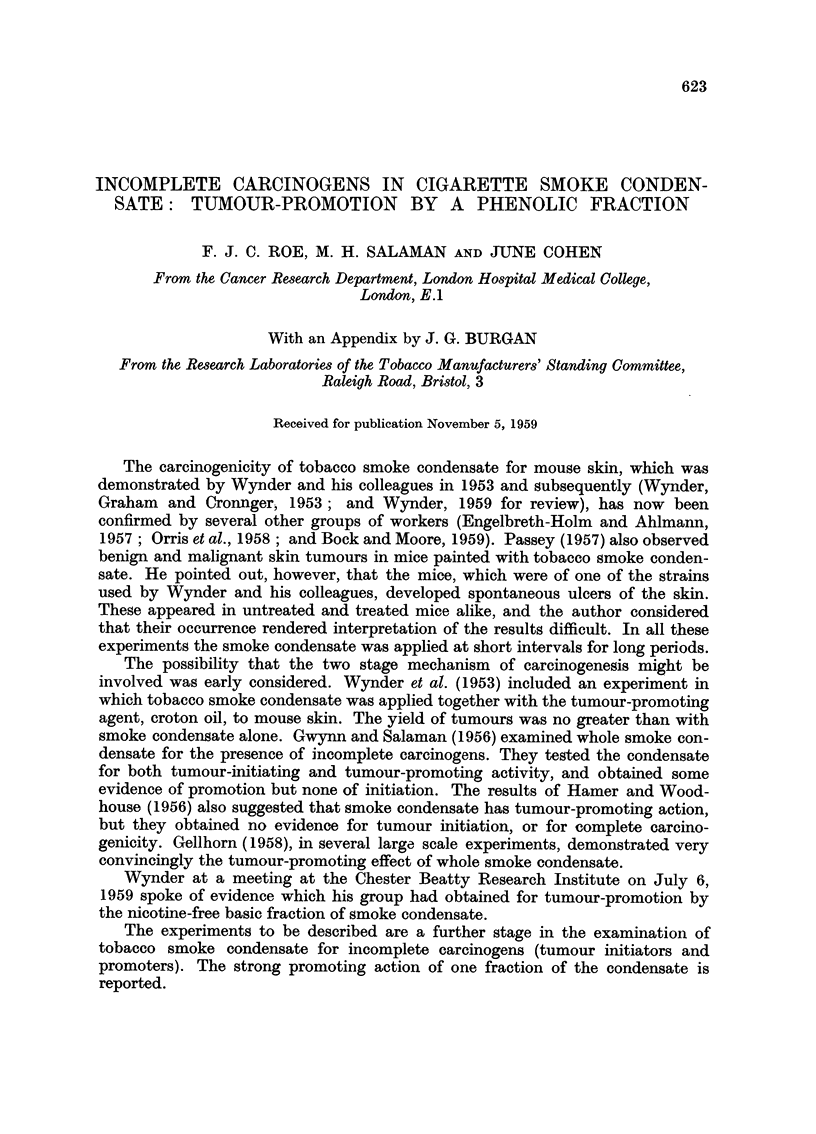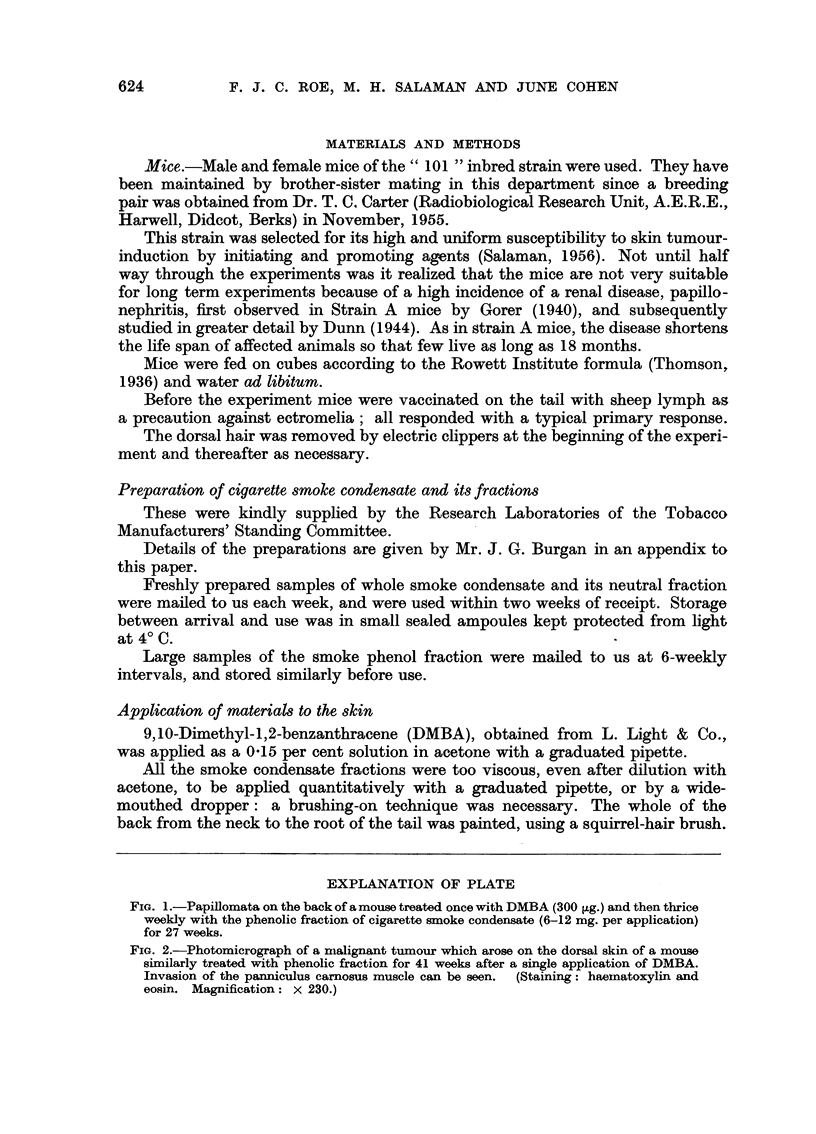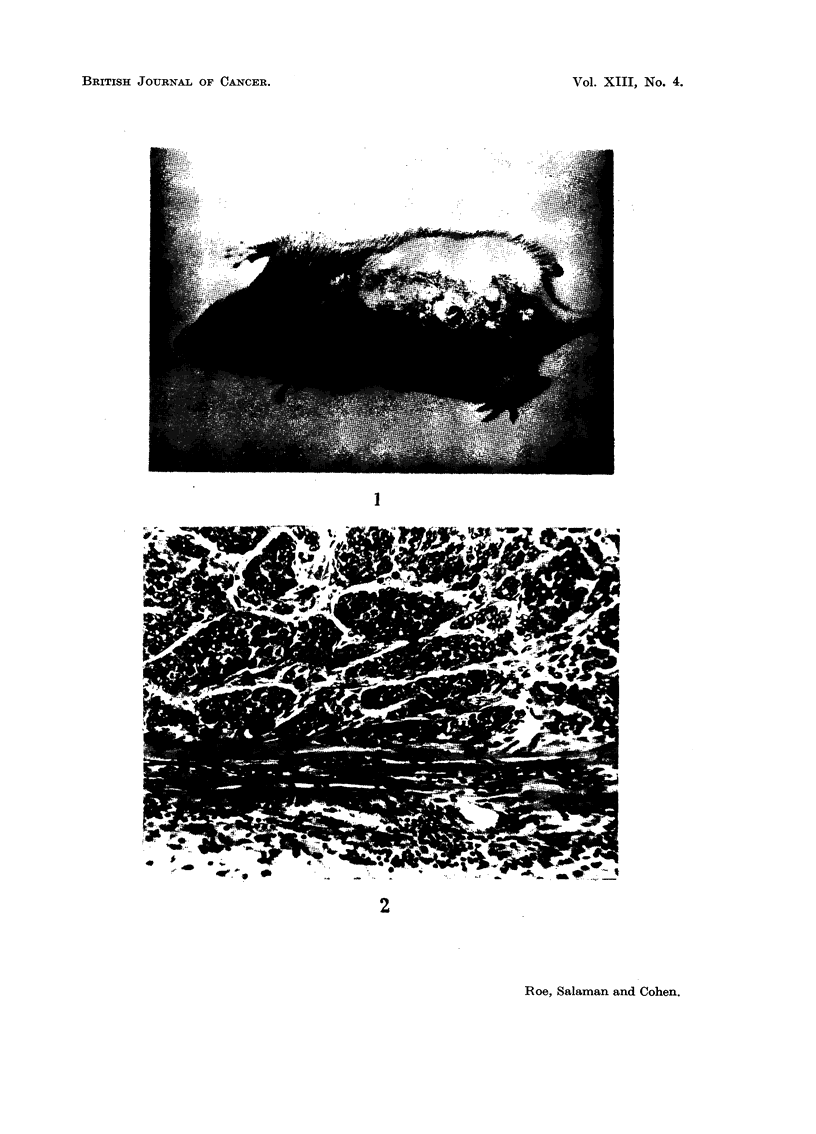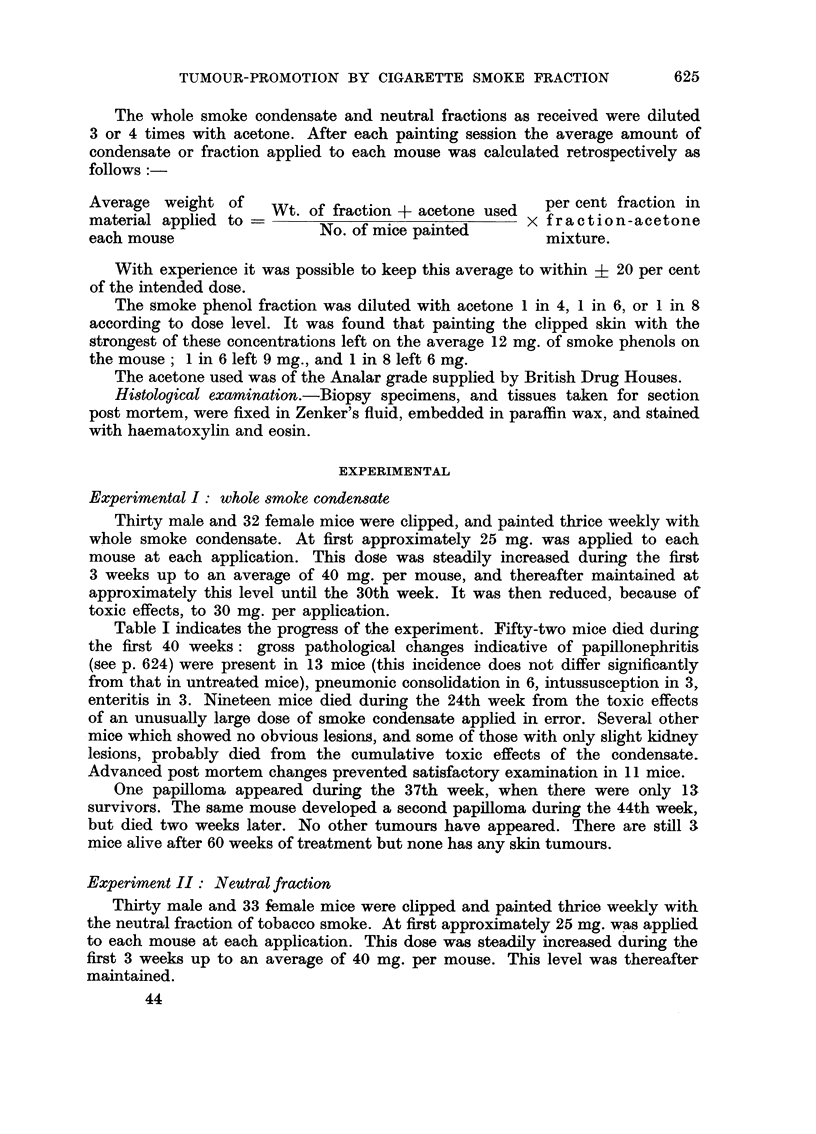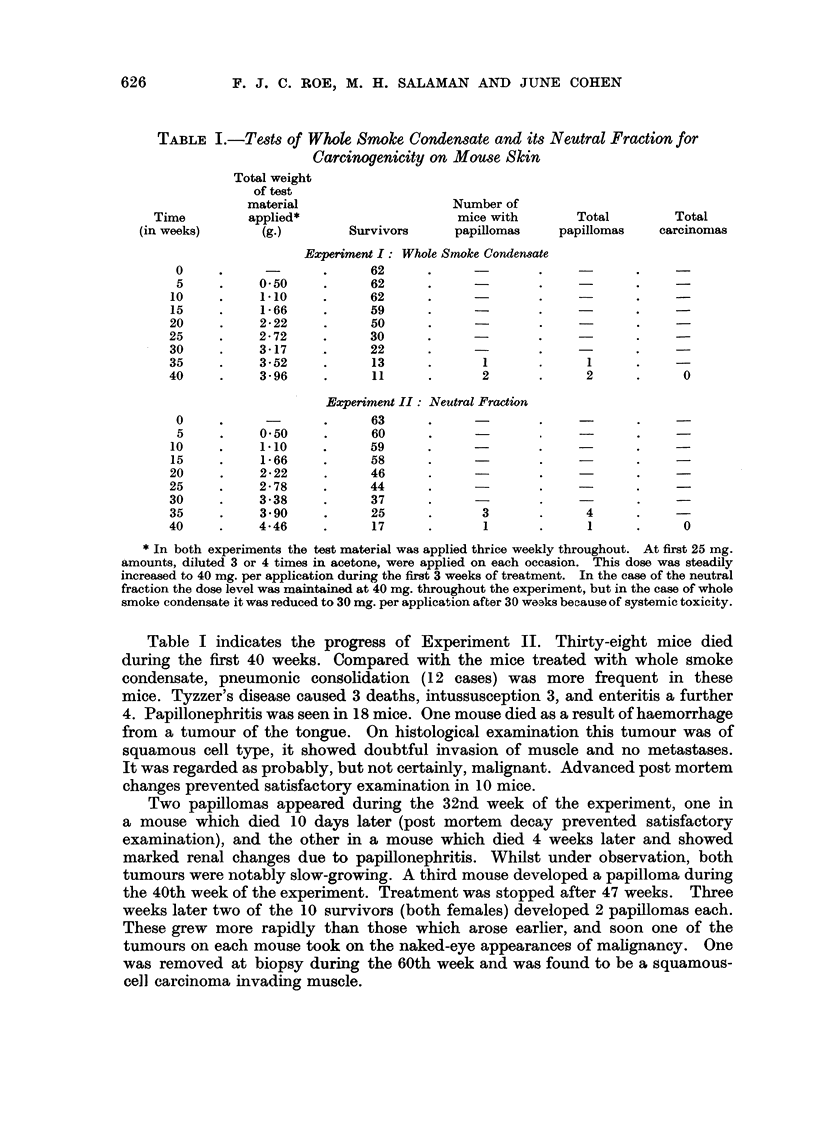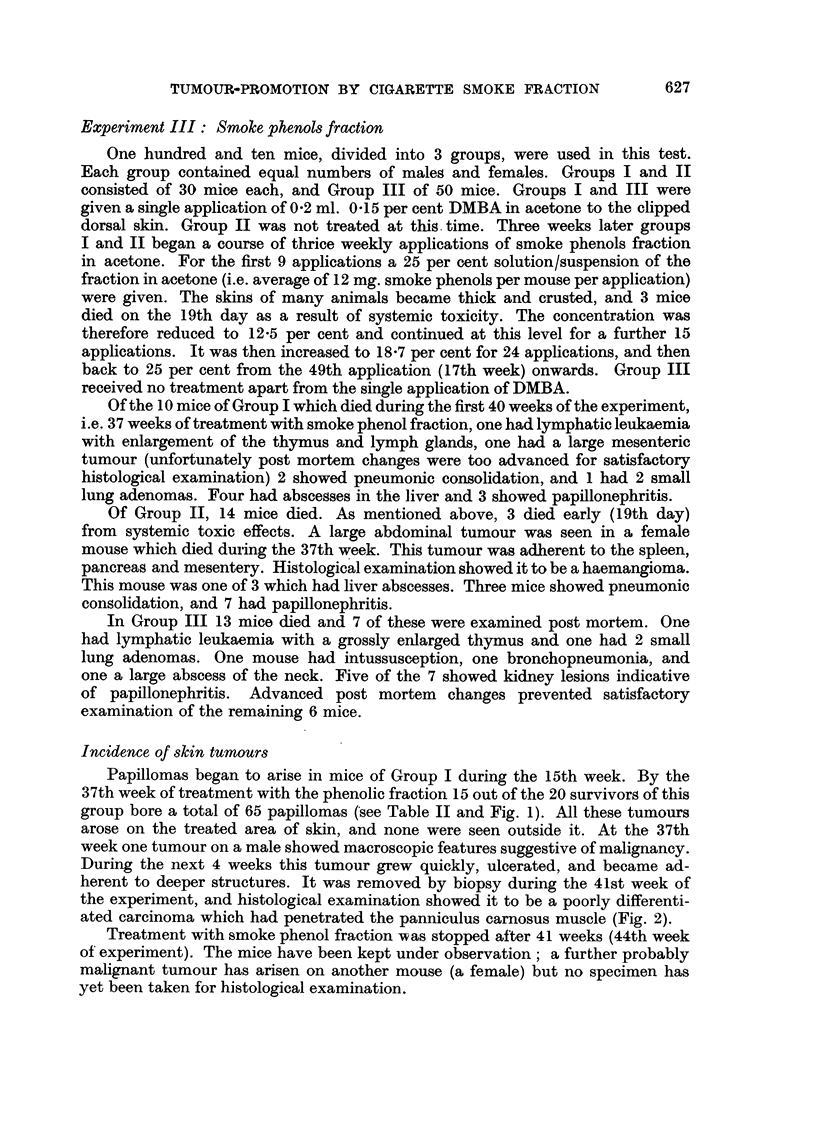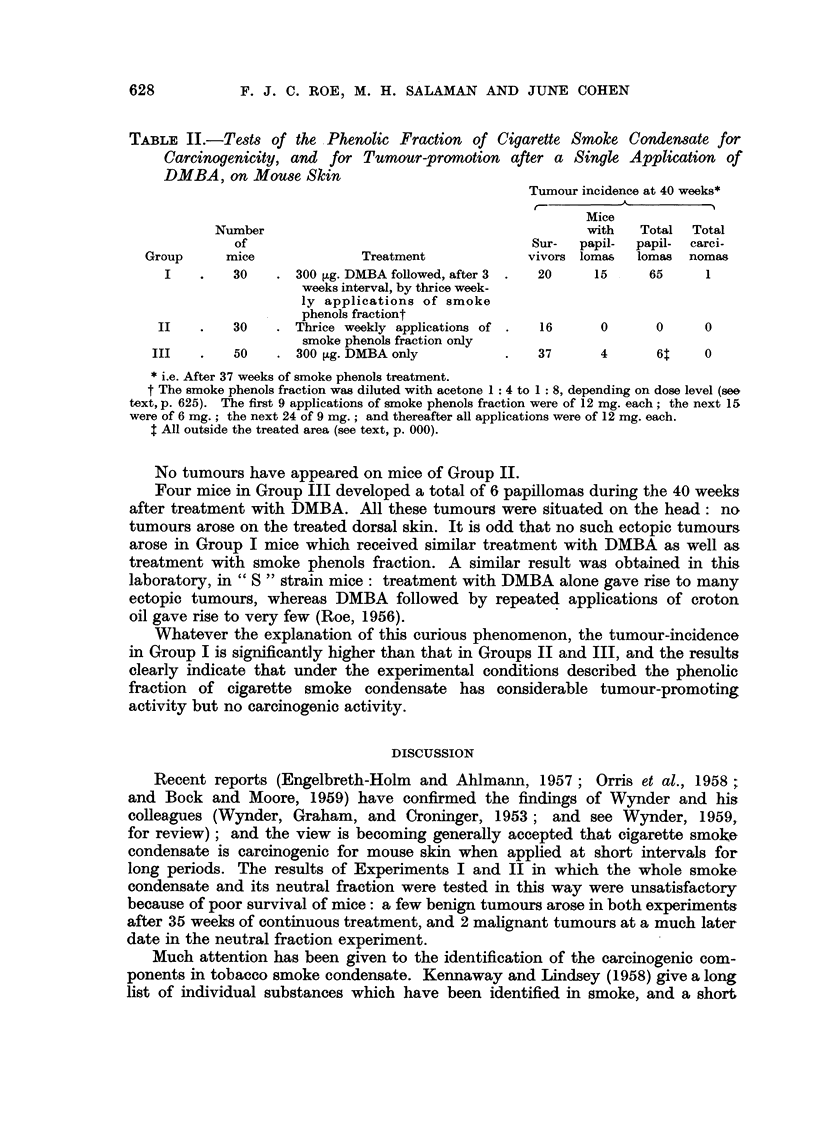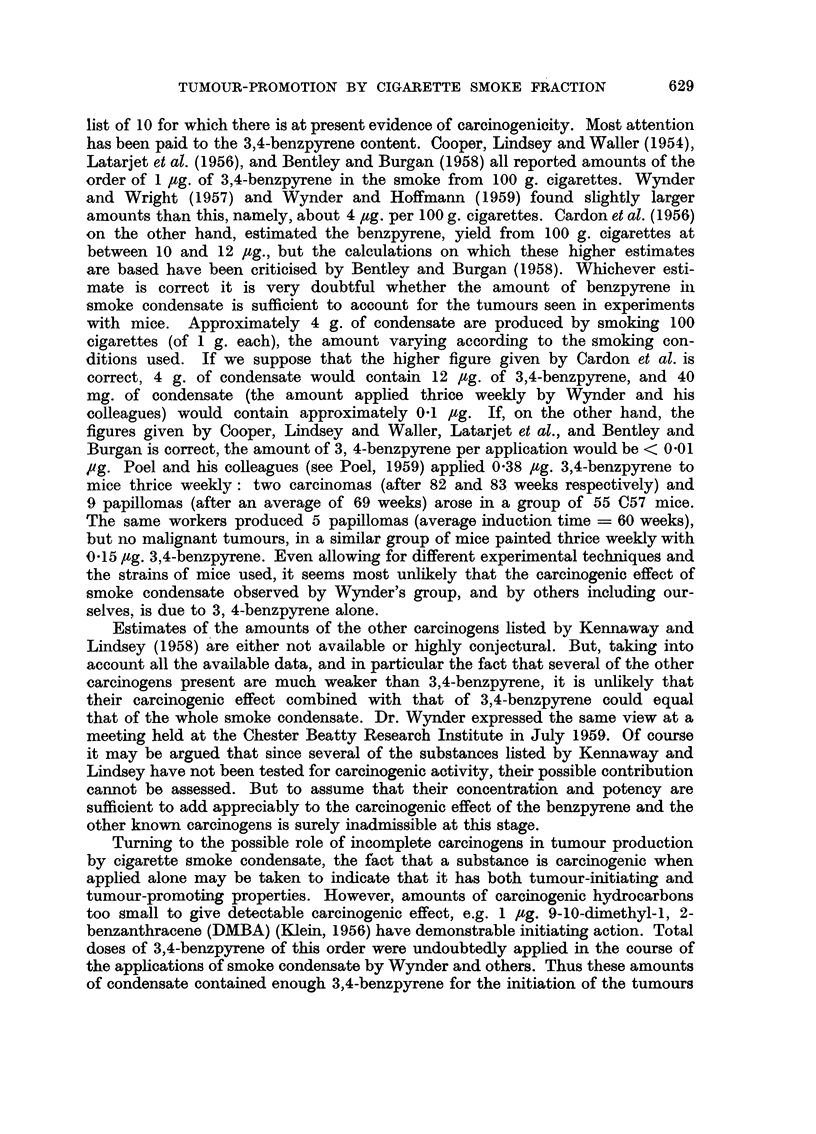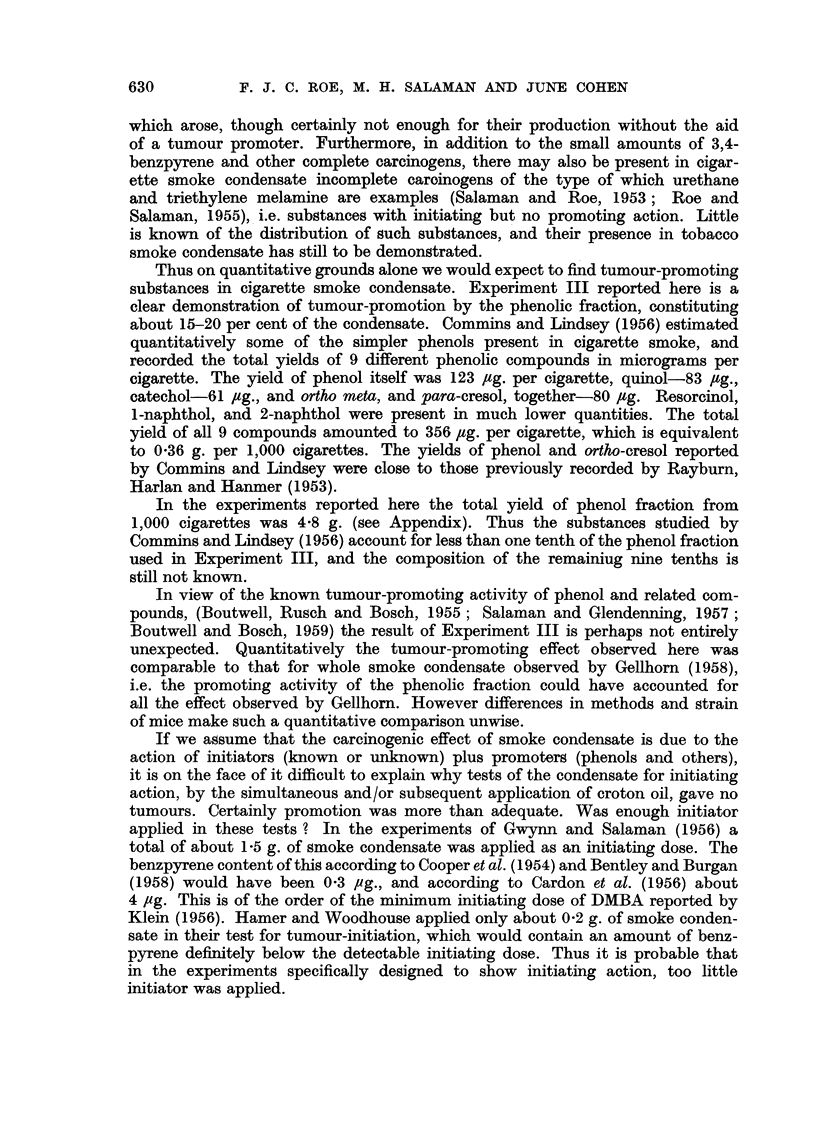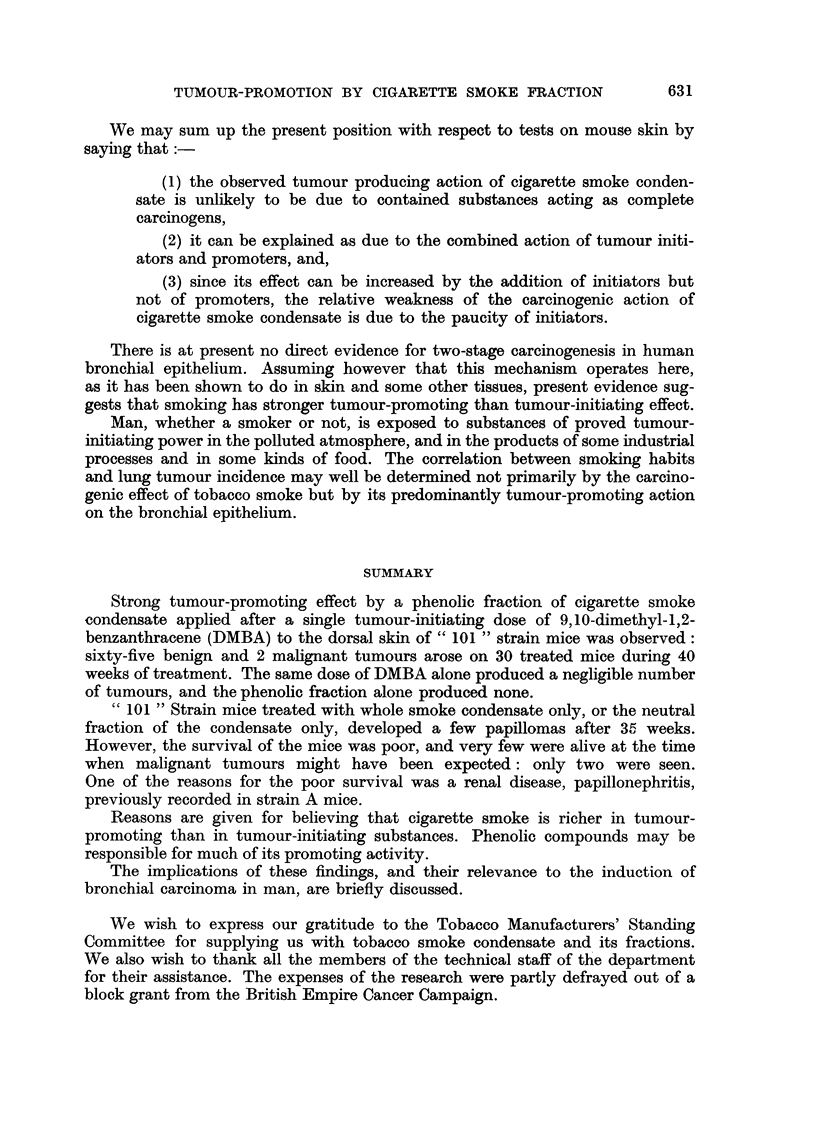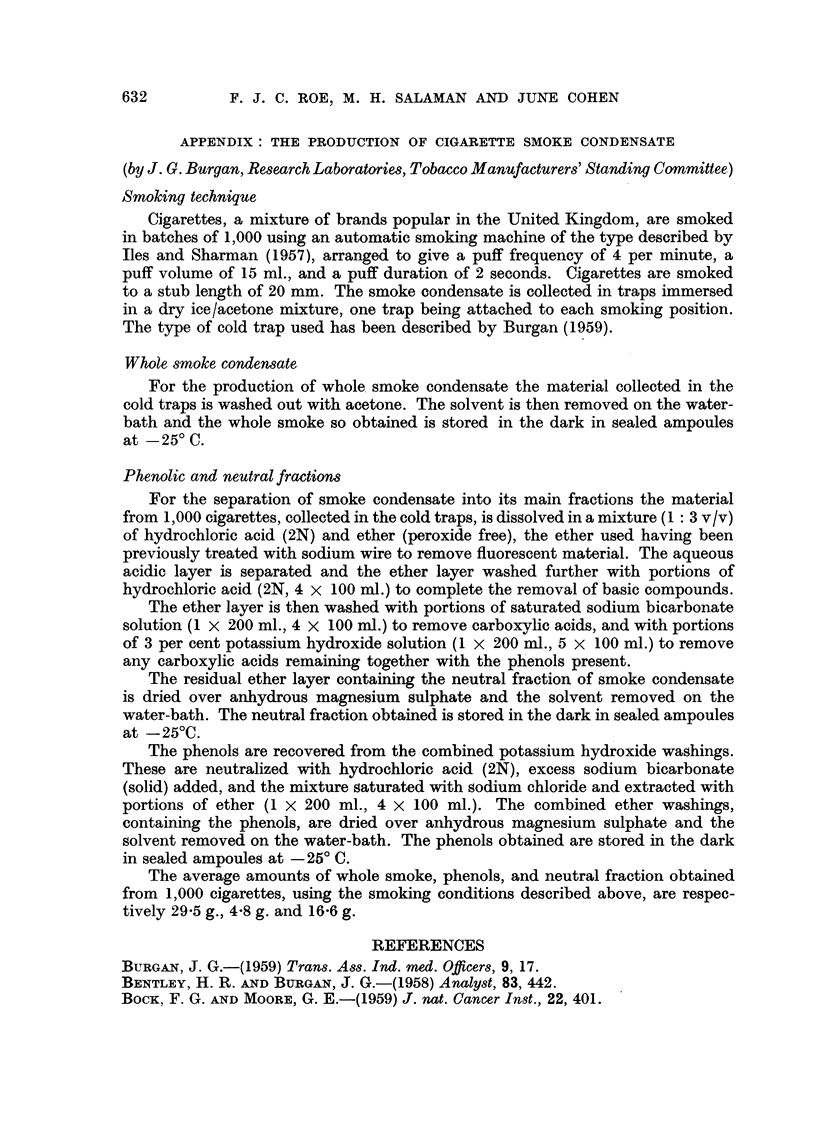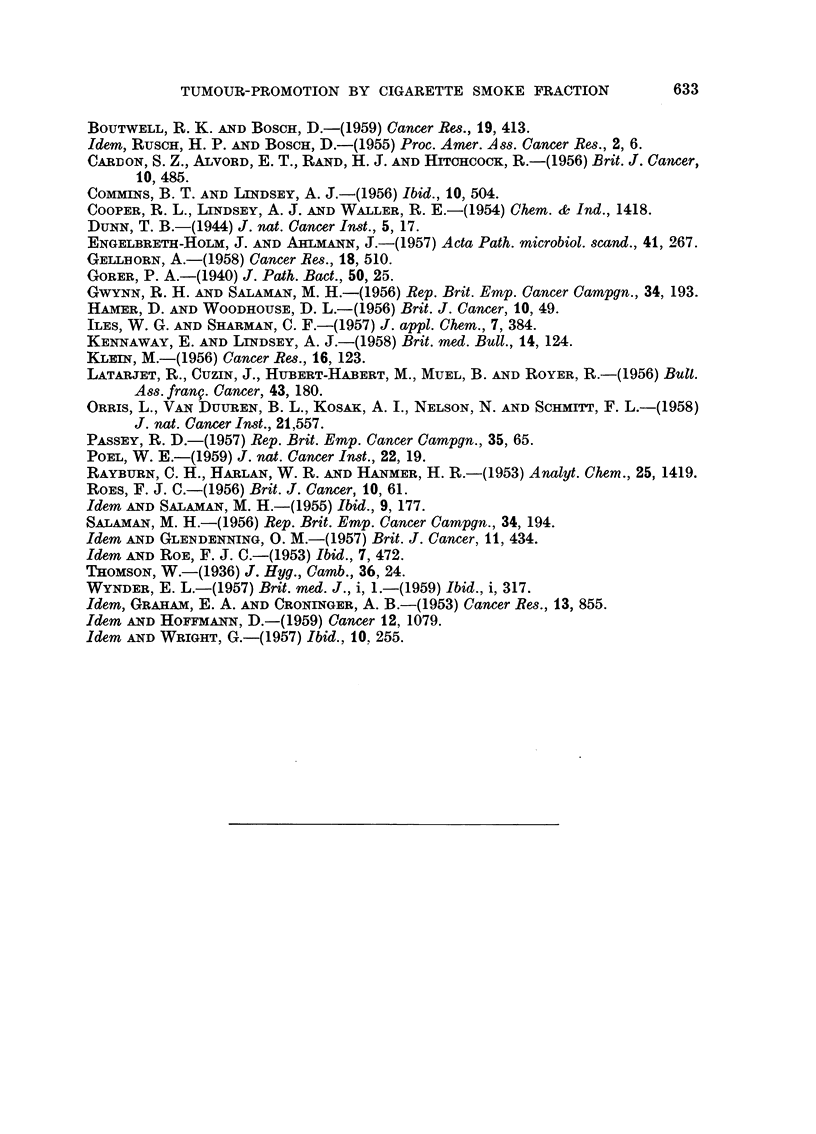# Incomplete Carcinogens in Cigarette Smoke Condensate: Tumour-Promotion by a Phenolic Fraction

**DOI:** 10.1038/bjc.1959.68

**Published:** 1959-12

**Authors:** F. J. C. Roe, M. H. Salaman, June Cohen

## Abstract

**Images:**


### INCOMPLETE CARCINOGENS IN CIGARETTE SMOKE CONDENSATE: TUMOUR-PROMOTION BY A PHENOLIC FRACTION

F. J. C. ROE, M. H. SALAMAN AND JUNE COHEN

*From the Cancer Research Department, London Hospital Medical College, London, E.*1

With an Appendix by J. G. BURGAN

*From the Research Laboratories of the Tobacco Manufacturers' Standing Committee, Raleigh Road, Bristol,* 3

Received for publication November 5, 1959

The carcinogenicity of tobacco smoke condensate for mouse skin, which was demonstrated by Wynder and his colleagues in 1953 and subsequently (Wynder, Graham and Cronnger, 1953; and Wynder, 1959 for review), has now been confirmed by several other groups of workers (Engelbreth-Holm and Ahlmann, 1957; Orris *et al.*, 1958; and Bock and Moore, 1959). Passey (1957) also observed benign and malignant skin tumours in mice painted with tobacco smoke condensate. He pointed out, however, that the mice, which were of one of the strains used by Wynder and his colleagues, developed spontaneous ulcers of the skin. These appeared in untreated and treated mice alike, and the author considered that their occurrence rendered interpretation of the results difficult. In all these experiments the smoke condensate was applied at short intervals for long periods.

The possibility that the two stage mechanism of carcinogenesis might be involved was early considered. Wynder *et al.* (1953) included an experiment in which tobacco smoke condensate was applied together with the tumour-promoting agent, croton oil, to mouse skin. The yield of tumours was no greater than with smoke condensate alone. Gwynn and Salaman (1956) examined whole smoke condensate for the presence of incomplete carcinogens. They tested the condensate for both tumour-initiating and tumour-promoting activity, and obtained some evidence of promotion but none of initiation. The results of Hamer and Woodhouse (1956) also suggested that smoke condensate has tumour-promoting action, but they obtained no evidence for tumour initiation, or for complete carcinogenicity. Gellhorn (1958), in several large scale experiments, demonstrated very convincingly the tumour-promoting effect of whole smoke condensate.

Wynder at a meeting at the Chester Beatty Research Institute on July 6, 1959 spoke of evidence which his group had obtained for tumour-promotion by the nicotine-free basic fraction of smoke condensate.

The experiments to be described are a further stage in the examination of tobacco smoke condensate for incomplete carcinogens (tumour initiators and promoters). The strong promoting action of one fraction of the condensate is reported.

### MATERIALS AND METHODS

*Mice.*—Male and female mice of the " 101 " inbred strain were used. They have been maintained by brother-sister mating in this department since a breeding pair was obtained from Dr. T. C. Carter (Radiobiological Research Unit, A.E.R.E., Harwell, Didcot, Berks) in November, 1955.

This strain was selected for its high and uniform susceptibility to skin tumour-induction by initiating and promoting agents (Salaman, 1956). Not until half way through the experiments was it realized that the mice are not very suitable for long term experiments because of a high incidence of a renal disease, papillo-nephritis, first observed in Strain A mice by Gorer (1940), and subsequently studied in greater detail by Dunn (1944). As in strain A mice, the disease shortens the life span of affected animals so that few live as long as 18 months.

Mice were fed on cubes according to the Rowett Institute formula (Thomson, 1936) and water *ad libitum.*

Before the experiment mice were vaccinated on the tail with sheep lymph as a precaution against ectromelia ; all responded with a typical primary response.

The dorsal hair was removed by electric clippers at the beginning of the experiment and thereafter as necessary.

### *Preparation of cigarette smoke condensate and its fractions*

These were kindly supplied by the Research Laboratories of the Tobacco Manufacturers' Standing Committee.

Details of the preparations are given by Mr. J. G. Burgan in an appendix to this paper.

Freshly prepared samples of whole smoke condensate and its neutral fraction were mailed to us each week, and were used within two weeks of receipt. Storage between arrival and use was in small sealed ampoules kept protected from light at 4° C.

Large samples of the smoke phenol fraction were mailed to us at 6-weekly intervals, and stored similarly before use.

### *Application of materials to the skin*

9,10-Dimethyl-1,2-benzanthracene (DMBA), obtained from L. Light & Co., was applied as a 0·15 per cent solution in acetone with a graduated pipette.

All the smoke condensate fractions were too viscous, even after dilution with acetone, to be applied quantitatively with a graduated pipette, or by a wide-mouthed dropper : a brushing-on technique was necessary. The whole of the back from the neck to the root of the tail was painted, using a squirrel-hair brush.

---

EXPLANATION OF PLATE

FIG. 1.—Papillomata on the back of a mouse treated once with DMBA (300 μg.) and then thrice weekly with the phenolic fraction of cigarette smoke condensate (6–12 mg. per application) for 27 weeks.

FIG. 2.—Photomicrograph of a malignant tumour which arose on the dorsal skin of a mouse similarly treated with phenolic fraction for 41 weeks after a single application of DMBA. Invasion of the panniculus carnosus muscle can be seen. (Staining : haematoxylin and eosin. Magnification : × 230.)

1

2

Roe, Salaman and Cohen.

The whole smoke condensate and neutral fractions as received were diluted 3 or 4 times with acetone. After each painting session the average amount of condensate or fraction applied to each mouse was calculated retrospectively as follows :—

$$\text{Average weight of material applied to each mouse} = \frac{\text{Wt. of fraction} + \text{acetone used}}{\text{No. of mice painted}} \times \text{per cent fraction in fraction-acetone mixture.}$$

With experience it was possible to keep this average to within $\pm$ 20 per cent of the intended dose.

The smoke phenol fraction was diluted with acetone 1 in 4, 1 in 6, or 1 in 8 according to dose level. It was found that painting the clipped skin with the strongest of these concentrations left on the average 12 mg. of smoke phenols on the mouse ; 1 in 6 left 9 mg., and 1 in 8 left 6 mg.

The acetone used was of the Analar grade supplied by British Drug Houses.

*Histological examination.*—Biopsy specimens, and tissues taken for section post mortem, were fixed in Zenker's fluid, embedded in paraffin wax, and stained with haematoxylin and eosin.

### EXPERIMENTAL

### *Experimental I : whole smoke condensate*

Thirty male and 32 female mice were clipped, and painted thrice weekly with whole smoke condensate. At first approximately 25 mg. was applied to each mouse at each application. This dose was steadily increased during the first 3 weeks up to an average of 40 mg. per mouse, and thereafter maintained at approximately this level until the 30th week. It was then reduced, because of toxic effects, to 30 mg. per application.

Table I indicates the progress of the experiment. Fifty-two mice died during the first 40 weeks : gross pathological changes indicative of papillonephritis (see p. 624) were present in 13 mice (this incidence does not differ significantly from that in untreated mice), pneumonic consolidation in 6, intussusception in 3, enteritis in 3. Nineteen mice died during the 24th week from the toxic effects of an unusually large dose of smoke condensate applied in error. Several other mice which showed no obvious lesions, and some of those with only slight kidney lesions, probably died from the cumulative toxic effects of the condensate. Advanced post mortem changes prevented satisfactory examination in 11 mice.

One papilloma appeared during the 37th week, when there were only 13 survivors. The same mouse developed a second papilloma during the 44th week, but died two weeks later. No other tumours have appeared. There are still 3 mice alive after 60 weeks of treatment but none has any skin tumours.

### *Experiment II : Neutral fraction*

Thirty male and 33 female mice were clipped and painted thrice weekly with the neutral fraction of tobacco smoke. At first approximately 25 mg. was applied to each mouse at each application. This dose was steadily increased during the first 3 weeks up to an average of 40 mg. per mouse. This level was thereafter maintained.

TABLE I.—*Tests of Whole Smoke Condensate and its Neutral Fraction for Carcinogenicity on Mouse Skin*

| Time (in weeks) | Total weight of test material applied* (g.) | Survivors | Number of mice with papillomas | Total papillomas | Total carcinomas |
|---|---|---|---|---|---|
| | | *Experiment I : Whole Smoke Condensate* | | | |
| 0 | — | 62 | — | — | — |
| 5 | 0·50 | 62 | — | — | — |
| 10 | 1·10 | 62 | — | — | — |
| 15 | 1·66 | 59 | — | — | — |
| 20 | 2·22 | 50 | — | — | — |
| 25 | 2·72 | 30 | — | — | — |
| 30 | 3·17 | 22 | — | — | — |
| 35 | 3·52 | 13 | 1 | 1 | — |
| 40 | 3·96 | 11 | 2 | 2 | 0 |
| | | *Experiment II : Neutral Fraction* | | | |
| 0 | — | 63 | — | — | — |
| 5 | 0·50 | 60 | — | — | — |
| 10 | 1·10 | 59 | — | — | — |
| 15 | 1·66 | 58 | — | — | — |
| 20 | 2·22 | 46 | — | — | — |
| 25 | 2·78 | 44 | — | — | — |
| 30 | 3·38 | 37 | — | — | — |
| 35 | 3·90 | 25 | 3 | 4 | — |
| 40 | 4·46 | 17 | 1 | 1 | 0 |

* In both experiments the test material was applied thrice weekly throughout. At first 25 mg. amounts, diluted 3 or 4 times in acetone, were applied on each occasion. This dose was steadily increased to 40 mg. per application during the first 3 weeks of treatment. In the case of the neutral fraction the dose level was maintained at 40 mg. throughout the experiment, but in the case of whole smoke condensate it was reduced to 30 mg. per application after 30 weeks because of systemic toxicity.

Table I indicates the progress of Experiment II. Thirty-eight mice died during the first 40 weeks. Compared with the mice treated with whole smoke condensate, pneumonic consolidation (12 cases) was more frequent in these mice. Tyzzer's disease caused 3 deaths, intussusception 3, and enteritis a further 4. Papillonephritis was seen in 18 mice. One mouse died as a result of haemorrhage from a tumour of the tongue. On histological examination this tumour was of squamous cell type, it showed doubtful invasion of muscle and no metastases. It was regarded as probably, but not certainly, malignant. Advanced post mortem changes prevented satisfactory examination in 10 mice.

Two papillomas appeared during the 32nd week of the experiment, one in a mouse which died 10 days later (post mortem decay prevented satisfactory examination), and the other in a mouse which died 4 weeks later and showed marked renal changes due to papillonephritis. Whilst under observation, both tumours were notably slow-growing. A third mouse developed a papilloma during the 40th week of the experiment. Treatment was stopped after 47 weeks. Three weeks later two of the 10 survivors (both females) developed 2 papillomas each. These grew more rapidly than those which arose earlier, and soon one of the tumours on each mouse took on the naked-eye appearances of malignancy. One was removed at biopsy during the 60th week and was found to be a squamous-cell carcinoma invading muscle.

*Experiment III : Smoke phenols fraction*

One hundred and ten mice, divided into 3 groups, were used in this test. Each group contained equal numbers of males and females. Groups I and II consisted of 30 mice each, and Group III of 50 mice. Groups I and III were given a single application of 0·2 ml. 0·15 per cent DMBA in acetone to the clipped dorsal skin. Group II was not treated at this time. Three weeks later groups I and II began a course of thrice weekly applications of smoke phenols fraction in acetone. For the first 9 applications a 25 per cent solution/suspension of the fraction in acetone (i.e. average of 12 mg. smoke phenols per mouse per application) were given. The skins of many animals became thick and crusted, and 3 mice died on the 19th day as a result of systemic toxicity. The concentration was therefore reduced to 12·5 per cent and continued at this level for a further 15 applications. It was then increased to 18·7 per cent for 24 applications, and then back to 25 per cent from the 49th application (17th week) onwards. Group III received no treatment apart from the single application of DMBA.

Of the 10 mice of Group I which died during the first 40 weeks of the experiment, i.e. 37 weeks of treatment with smoke phenol fraction, one had lymphatic leukaemia with enlargement of the thymus and lymph glands, one had a large mesenteric tumour (unfortunately post mortem changes were too advanced for satisfactory histological examination) 2 showed pneumonic consolidation, and 1 had 2 small lung adenomas. Four had abscesses in the liver and 3 showed papillonephritis.

Of Group II, 14 mice died. As mentioned above, 3 died early (19th day) from systemic toxic effects. A large abdominal tumour was seen in a female mouse which died during the 37th week. This tumour was adherent to the spleen, pancreas and mesentery. Histological examination showed it to be a haemangioma. This mouse was one of 3 which had liver abscesses. Three mice showed pneumonic consolidation, and 7 had papillonephritis.

In Group III 13 mice died and 7 of these were examined post mortem. One had lymphatic leukaemia with a grossly enlarged thymus and one had 2 small lung adenomas. One mouse had intussusception, one bronchopneumonia, and one a large abscess of the neck. Five of the 7 showed kidney lesions indicative of papillonephritis. Advanced post mortem changes prevented satisfactory examination of the remaining 6 mice.

*Incidence of skin tumours*

Papillomas began to arise in mice of Group I during the 15th week. By the 37th week of treatment with the phenolic fraction 15 out of the 20 survivors of this group bore a total of 65 papillomas (see Table II and Fig. 1). All these tumours arose on the treated area of skin, and none were seen outside it. At the 37th week one tumour on a male showed macroscopic features suggestive of malignancy. During the next 4 weeks this tumour grew quickly, ulcerated, and became adherent to deeper structures. It was removed by biopsy during the 41st week of the experiment, and histological examination showed it to be a poorly differentiated carcinoma which had penetrated the panniculus carnosus muscle (Fig. 2).

Treatment with smoke phenol fraction was stopped after 41 weeks (44th week of experiment). The mice have been kept under observation ; a further probably malignant tumour has arisen on another mouse (a female) but no specimen has yet been taken for histological examination.

TABLE II.—*Tests of the Phenolic Fraction of Cigarette Smoke Condensate for Carcinogenicity, and for Tumour-promotion after a Single Application of DMBA, on Mouse Skin*

| | | | Tumour incidence at 40 weeks* | | | |
|---|---|---|---|---|---|---|
| Group | Number of mice | Treatment | Survivors | Mice with papillomas | Total papillomas | Total carcinomas |
| I | 30 | 300 μg. DMBA followed, after 3 weeks interval, by thrice weekly applications of smoke phenols fraction† | 20 | 15 | 65 | 1 |
| II | 30 | Thrice weekly applications of smoke phenols fraction only | 16 | 0 | 0 | 0 |
| III | 50 | 300 μg. DMBA only | 37 | 4 | 6‡ | 0 |

* i.e. After 37 weeks of smoke phenols treatment.

† The smoke phenols fraction was diluted with acetone 1 : 4 to 1 : 8, depending on dose level (see text, p. 625). The first 9 applications of smoke phenols fraction were of 12 mg. each ; the next 15 were of 6 mg. ; the next 24 of 9 mg. ; and thereafter all applications were of 12 mg. each.

‡ All outside the treated area (see text, p. 000).

No tumours have appeared on mice of Group II.

Four mice in Group III developed a total of 6 papillomas during the 40 weeks after treatment with DMBA. All these tumours were situated on the head : no tumours arose on the treated dorsal skin. It is odd that no such ectopic tumours arose in Group I mice which received similar treatment with DMBA as well as treatment with smoke phenols fraction. A similar result was obtained in this laboratory, in " S " strain mice : treatment with DMBA alone gave rise to many ectopic tumours, whereas DMBA followed by repeated applications of croton oil gave rise to very few (Roe, 1956).

Whatever the explanation of this curious phenomenon, the tumour-incidence in Group I is significantly higher than that in Groups II and III, and the results clearly indicate that under the experimental conditions described the phenolic fraction of cigarette smoke condensate has considerable tumour-promoting activity but no carcinogenic activity.

### DISCUSSION

Recent reports (Engelbreth-Holm and Ahlmann, 1957 ; Orris *et al.*, 1958 ; and Bock and Moore, 1959) have confirmed the findings of Wynder and his colleagues (Wynder, Graham, and Croninger, 1953 ; and see Wynder, 1959, for review) ; and the view is becoming generally accepted that cigarette smoke condensate is carcinogenic for mouse skin when applied at short intervals for long periods. The results of Experiments I and II in which the whole smoke condensate and its neutral fraction were tested in this way were unsatisfactory because of poor survival of mice : a few benign tumours arose in both experiments after 35 weeks of continuous treatment, and 2 malignant tumours at a much later date in the neutral fraction experiment.

Much attention has been given to the identification of the carcinogenic components in tobacco smoke condensate. Kennaway and Lindsey (1958) give a long list of individual substances which have been identified in smoke, and a short list of 10 for which there is at present evidence of carcinogenicity. Most attention has been paid to the 3,4-benzpyrene content. Cooper, Lindsey and Waller (1954), Latarjet *et al.* (1956), and Bentley and Burgan (1958) all reported amounts of the order of 1 $\mu$g. of 3,4-benzpyrene in the smoke from 100 g. cigarettes. Wynder and Wright (1957) and Wynder and Hoffmann (1959) found slightly larger amounts than this, namely, about 4 $\mu$g. per 100 g. cigarettes. Cardon *et al.* (1956) on the other hand, estimated the benzpyrene, yield from 100 g. cigarettes at between 10 and 12 $\mu$g., but the calculations on which these higher estimates are based have been criticised by Bentley and Burgan (1958). Whichever estimate is correct it is very doubtful whether the amount of benzpyrene in smoke condensate is sufficient to account for the tumours seen in experiments with mice. Approximately 4 g. of condensate are produced by smoking 100 cigarettes (of 1 g. each), the amount varying according to the smoking conditions used. If we suppose that the higher figure given by Cardon *et al.* is correct, 4 g. of condensate would contain 12 $\mu$g. of 3,4-benzpyrene, and 40 mg. of condensate (the amount applied thrice weekly by Wynder and his colleagues) would contain approximately 0·1 $\mu$g. If, on the other hand, the figures given by Cooper, Lindsey and Waller, Latarjet *et al.*, and Bentley and Burgan is correct, the amount of 3, 4-benzpyrene per application would be $<$ 0·01 $\mu$g. Poel and his colleagues (see Poel, 1959) applied 0·38 $\mu$g. 3,4-benzpyrene to mice thrice weekly: two carcinomas (after 82 and 83 weeks respectively) and 9 papillomas (after an average of 69 weeks) arose in a group of 55 C57 mice. The same workers produced 5 papillomas (average induction time = 60 weeks), but no malignant tumours, in a similar group of mice painted thrice weekly with 0·15 $\mu$g. 3,4-benzpyrene. Even allowing for different experimental techniques and the strains of mice used, it seems most unlikely that the carcinogenic effect of smoke condensate observed by Wynder's group, and by others including ourselves, is due to 3, 4-benzpyrene alone.

Estimates of the amounts of the other carcinogens listed by Kennaway and Lindsey (1958) are either not available or highly conjectural. But, taking into account all the available data, and in particular the fact that several of the other carcinogens present are much weaker than 3,4-benzpyrene, it is unlikely that their carcinogenic effect combined with that of 3,4-benzpyrene could equal that of the whole smoke condensate. Dr. Wynder expressed the same view at a meeting held at the Chester Beatty Research Institute in July 1959. Of course it may be argued that since several of the substances listed by Kennaway and Lindsey have not been tested for carcinogenic activity, their possible contribution cannot be assessed. But to assume that their concentration and potency are sufficient to add appreciably to the carcinogenic effect of the benzpyrene and the other known carcinogens is surely inadmissible at this stage.

Turning to the possible role of incomplete carcinogens in tumour production by cigarette smoke condensate, the fact that a substance is carcinogenic when applied alone may be taken to indicate that it has both tumour-initiating and tumour-promoting properties. However, amounts of carcinogenic hydrocarbons too small to give detectable carcinogenic effect, e.g. 1 $\mu$g. 9-10-dimethyl-1, 2-benzanthracene (DMBA) (Klein, 1956) have demonstrable initiating action. Total doses of 3,4-benzpyrene of this order were undoubtedly applied in the course of the applications of smoke condensate by Wynder and others. Thus these amounts of condensate contained enough 3,4-benzpyrene for the initiation of the tumours which arose, though certainly not enough for their production without the aid of a tumour promoter. Furthermore, in addition to the small amounts of 3,4-benzpyrene and other complete carcinogens, there may also be present in cigarette smoke condensate incomplete carcinogens of the type of which urethane and triethylene melamine are examples (Salaman and Roe, 1953; Roe and Salaman, 1955), i.e. substances with initiating but no promoting action. Little is known of the distribution of such substances, and their presence in tobacco smoke condensate has still to be demonstrated.

Thus on quantitative grounds alone we would expect to find tumour-promoting substances in cigarette smoke condensate. Experiment III reported here is a clear demonstration of tumour-promotion by the phenolic fraction, constituting about 15–20 per cent of the condensate. Commins and Lindsey (1956) estimated quantitatively some of the simpler phenols present in cigarette smoke, and recorded the total yields of 9 different phenolic compounds in micrograms per cigarette. The yield of phenol itself was 123 $\mu$g. per cigarette, quinol—83 $\mu$g., catechol—61 $\mu$g., and *ortho meta*, and *para*-cresol, together—80 $\mu$g. Resorcinol, 1-naphthol, and 2-naphthol were present in much lower quantities. The total yield of all 9 compounds amounted to 356 $\mu$g. per cigarette, which is equivalent to 0·36 g. per 1,000 cigarettes. The yields of phenol and *ortho*-cresol reported by Commins and Lindsey were close to those previously recorded by Rayburn, Harlan and Hanmer (1953).

In the experiments reported here the total yield of phenol fraction from 1,000 cigarettes was 4·8 g. (see Appendix). Thus the substances studied by Commins and Lindsey (1956) account for less than one tenth of the phenol fraction used in Experiment III, and the composition of the remainiug nine tenths is still not known.

In view of the known tumour-promoting activity of phenol and related compounds, (Boutwell, Rusch and Bosch, 1955; Salaman and Glendenning, 1957; Boutwell and Bosch, 1959) the result of Experiment III is perhaps not entirely unexpected. Quantitatively the tumour-promoting effect observed here was comparable to that for whole smoke condensate observed by Gellhorn (1958), i.e. the promoting activity of the phenolic fraction could have accounted for all the effect observed by Gellhorn. However differences in methods and strain of mice make such a quantitative comparison unwise.

If we assume that the carcinogenic effect of smoke condensate is due to the action of initiators (known or unknown) plus promoters (phenols and others), it is on the face of it difficult to explain why tests of the condensate for initiating action, by the simultaneous and/or subsequent application of croton oil, gave no tumours. Certainly promotion was more than adequate. Was enough initiator applied in these tests? In the experiments of Gwynn and Salaman (1956) a total of about 1·5 g. of smoke condensate was applied as an initiating dose. The benzpyrene content of this according to Cooper *et al.* (1954) and Bentley and Burgan (1958) would have been 0·3 $\mu$g., and according to Cardon *et al.* (1956) about 4 $\mu$g. This is of the order of the minimum initiating dose of DMBA reported by Klein (1956). Hamer and Woodhouse applied only about 0·2 g. of smoke condensate in their test for tumour-initiation, which would contain an amount of benzpyrene definitely below the detectable initiating dose. Thus it is probable that in the experiments specifically designed to show initiating action, too little initiator was applied.

We may sum up the present position with respect to tests on mouse skin by saying that :—

(1) the observed tumour producing action of cigarette smoke condensate is unlikely to be due to contained substances acting as complete carcinogens,

(2) it can be explained as due to the combined action of tumour initiators and promoters, and,

(3) since its effect can be increased by the addition of initiators but not of promoters, the relative weakness of the carcinogenic action of cigarette smoke condensate is due to the paucity of initiators.

There is at present no direct evidence for two-stage carcinogenesis in human bronchial epithelium. Assuming however that this mechanism operates here, as it has been shown to do in skin and some other tissues, present evidence suggests that smoking has stronger tumour-promoting than tumour-initiating effect.

Man, whether a smoker or not, is exposed to substances of proved tumour-initiating power in the polluted atmosphere, and in the products of some industrial processes and in some kinds of food. The correlation between smoking habits and lung tumour incidence may well be determined not primarily by the carcinogenic effect of tobacco smoke but by its predominantly tumour-promoting action on the bronchial epithelium.

### SUMMARY

Strong tumour-promoting effect by a phenolic fraction of cigarette smoke condensate applied after a single tumour-initiating dose of 9,10-dimethyl-1,2-benzanthracene (DMBA) to the dorsal skin of " 101 " strain mice was observed: sixty-five benign and 2 malignant tumours arose on 30 treated mice during 40 weeks of treatment. The same dose of DMBA alone produced a negligible number of tumours, and the phenolic fraction alone produced none.

" 101 " Strain mice treated with whole smoke condensate only, or the neutral fraction of the condensate only, developed a few papillomas after 35 weeks. However, the survival of the mice was poor, and very few were alive at the time when malignant tumours might have been expected: only two were seen. One of the reasons for the poor survival was a renal disease, papillonephritis, previously recorded in strain A mice.

Reasons are given for believing that cigarette smoke is richer in tumour-promoting than in tumour-initiating substances. Phenolic compounds may be responsible for much of its promoting activity.

The implications of these findings, and their relevance to the induction of bronchial carcinoma in man, are briefly discussed.

We wish to express our gratitude to the Tobacco Manufacturers' Standing Committee for supplying us with tobacco smoke condensate and its fractions. We also wish to thank all the members of the technical staff of the department for their assistance. The expenses of the research were partly defrayed out of a block grant from the British Empire Cancer Campaign.

### APPENDIX : THE PRODUCTION OF CIGARETTE SMOKE CONDENSATE

(*by J. G. Burgan, Research Laboratories, Tobacco Manufacturers' Standing Committee*)

### *Smoking technique*

Cigarettes, a mixture of brands popular in the United Kingdom, are smoked in batches of 1,000 using an automatic smoking machine of the type described by Iles and Sharman (1957), arranged to give a puff frequency of 4 per minute, a puff volume of 15 ml., and a puff duration of 2 seconds. Cigarettes are smoked to a stub length of 20 mm. The smoke condensate is collected in traps immersed in a dry ice/acetone mixture, one trap being attached to each smoking position. The type of cold trap used has been described by Burgan (1959).

### *Whole smoke condensate*

For the production of whole smoke condensate the material collected in the cold traps is washed out with acetone. The solvent is then removed on the water-bath and the whole smoke so obtained is stored in the dark in sealed ampoules at $-25°$ C.

### *Phenolic and neutral fractions*

For the separation of smoke condensate into its main fractions the material from 1,000 cigarettes, collected in the cold traps, is dissolved in a mixture (1 : 3 v/v) of hydrochloric acid (2N) and ether (peroxide free), the ether used having been previously treated with sodium wire to remove fluorescent material. The aqueous acidic layer is separated and the ether layer washed further with portions of hydrochloric acid (2N, $4 \times 100$ ml.) to complete the removal of basic compounds.

The ether layer is then washed with portions of saturated sodium bicarbonate solution ($1 \times 200$ ml., $4 \times 100$ ml.) to remove carboxylic acids, and with portions of 3 per cent potassium hydroxide solution ($1 \times 200$ ml., $5 \times 100$ ml.) to remove any carboxylic acids remaining together with the phenols present.

The residual ether layer containing the neutral fraction of smoke condensate is dried over anhydrous magnesium sulphate and the solvent removed on the water-bath. The neutral fraction obtained is stored in the dark in sealed ampoules at $-25°$C.

The phenols are recovered from the combined potassium hydroxide washings. These are neutralized with hydrochloric acid (2N), excess sodium bicarbonate (solid) added, and the mixture saturated with sodium chloride and extracted with portions of ether ($1 \times 200$ ml., $4 \times 100$ ml.). The combined ether washings, containing the phenols, are dried over anhydrous magnesium sulphate and the solvent removed on the water-bath. The phenols obtained are stored in the dark in sealed ampoules at $-25°$ C.

The average amounts of whole smoke, phenols, and neutral fraction obtained from 1,000 cigarettes, using the smoking conditions described above, are respectively 29·5 g., 4·8 g. and 16·6 g.

### REFERENCES

Burgan, J. G.—(1959) *Trans. Ass. Ind. med. Officers*, **9**, 17.
Bentley, H. R. and Burgan, J. G.—(1958) *Analyst*, **83**, 442.
Bock, F. G. and Moore, G. E.—(1959) *J. nat. Cancer Inst.*, **22**, 401.

BOUTWELL, R. K. AND BOSCH, D.—(1959) *Cancer Res.*, **19**, 413.
*Idem*, RUSCH, H. P. AND BOSCH, D.—(1955) *Proc. Amer. Ass. Cancer Res.*, **2**, 6.
CARDON, S. Z., ALVORD, E. T., RAND, H. J. AND HITCHCOCK, R.—(1956) *Brit. J. Cancer*, **10**, 485.
COMMINS, B. T. AND LINDSEY, A. J.—(1956) *Ibid.*, **10**, 504.
COOPER, R. L., LINDSEY, A. J. AND WALLER, R. E.—(1954) *Chem. & Ind.*, 1418.
DUNN, T. B.—(1944) *J. nat. Cancer Inst.*, **5**, 17.
ENGELBRETH-HOLM, J. AND AHLMANN, J.—(1957) *Acta Path. microbiol. scand.*, **41**, 267.
GELLHORN, A.—(1958) *Cancer Res.*, **18**, 510.
GORER, P. A.—(1940) *J. Path. Bact.*, **50**, 25.
GWYNN, R. H. AND SALAMAN, M. H.—(1956) *Rep. Brit. Emp. Cancer Campgn.*, **34**, 193.
HAMER, D. AND WOODHOUSE, D. L.—(1956) *Brit. J. Cancer*, **10**, 49.
ILES, W. G. AND SHARMAN, C. F.—(1957) *J. appl. Chem.*, **7**, 384.
KENNAWAY, E. AND LINDSEY, A. J.—(1958) *Brit. med. Bull.*, **14**, 124.
KLEIN, M.—(1956) *Cancer Res.*, **16**, 123.
LATARJET, R., CUZIN, J., HUBERT-HABERT, M., MUEL, B. AND ROYER, R.—(1956) *Bull. Ass. franç. Cancer*, **43**, 180.
ORRIS, L., VAN DUUREN, B. L., KOSAK, A. I., NELSON, N. AND SCHMITT, F. L.—(1958) *J. nat. Cancer Inst.*, **21**,557.
PASSEY, R. D.—(1957) *Rep. Brit. Emp. Cancer Campgn.*, **35**, 65.
POEL, W. E.—(1959) *J. nat. Cancer Inst.*, **22**, 19.
RAYBURN, C. H., HARLAN, W. R. AND HANMER, H. R.—(1953) *Analyt. Chem.*, **25**, 1419.
ROES, F. J. C.—(1956) *Brit. J. Cancer*, **10**, 61.
*Idem* AND SALAMAN, M. H.—(1955) *Ibid.*, **9**, 177.
SALAMAN, M. H.—(1956) *Rep. Brit. Emp. Cancer Campgn.*, **34**, 194.
*Idem* AND GLENDENNING, O. M.—(1957) *Brit. J. Cancer*, **11**, 434.
*Idem* AND ROE, F. J. C.—(1953) *Ibid.*, **7**, 472.
THOMSON, W.—(1936) *J. Hyg., Camb.*, **36**, 24.
WYNDER, E. L.—(1957) *Brit. med. J.*, i, 1.—(1959) *Ibid.*, i, 317.
*Idem*, GRAHAM, E. A. AND CRONINGER, A. B.—(1953) *Cancer Res.*, **13**, 855.
*Idem* AND HOFFMANN, D.—(1959) *Cancer* **12**, 1079.
*Idem* AND WRIGHT, G.—(1957) *Ibid.*, **10**, 255.